# Comparing the 2021 Census to administrative data to better understand the population estimation challenge

**DOI:** 10.23889/ijpds.v8i2.2260

**Published:** 2023-09-14

**Authors:** Elizabeth Pereira, Zak Robertson, Ayesha Barnes, Ffion Jones, Sally Mylles, Chloe Pearce

**Affiliations:** 1 Office for Nation Statistics, London, United Kingdom

## Objectives

To analyse the linked dataset between England and Wales 2021 Census/Census Coverage Survey and the Demographic Index (DI). This is a rare and unique opportunity to make direct comparisons between Census and administrative data to provide evidence on improvements in the use of administrative data in population and migration statistics.

## Methods

The DI is a composite dataset made up of several linked administrative sources. The linked output is complex, made up of individual sources grouped into clusters. These were linked to the Census, using both automatic and clerical matching to obtain the highest matching quality possible. To carry out the analysis, a flagging strategy has been designed to enable analysts to form cuts of the linked dataset that are specific to their research needs. High level research questions were designed to compare the coverage and capture across the Census and administrative data.

## Results

Initial analysis has been completed and reviewed, including: comparing the coverage of the DI and Statistical Population Datasets (which use inclusion rules to approximate the usual resident population) to Census, comparing how Communal establishments are captured in the DI and Census and comparing the geography of those found in both the DI and the Census.

The results will inform the National Statistician’s 2023 Recommendation, but also the future delivery of transformed population and migration statistics. This includes:

- Providing insights to improve the DI’s linkage methodology
- The SPD’s construct
- Identifying the extent of over and under coverage in the SPD, allowing development of an estimation strategy to estimate the population using administrative data more accurately

## Conclusion

This opportunity to compare linked administrative and Census data enables analysis to provide unique insights on quality and coverage, to improve the use of administrative data to inform population estimates, provide evidence on the use of additional administrative sources and improve future linkages.

